# Enhanced recovery protocols for colorectal surgery and postoperative renal function: a retrospective review

**DOI:** 10.1186/s13741-017-0069-0

**Published:** 2017-09-21

**Authors:** Charles R. Horres, Mohamed A. Adam, Zhifei Sun, Julie K. Thacker, Richard E. Moon, Timothy E. Miller, Stuart A. Grant

**Affiliations:** 1 Department of Anesthesiology, Duke University, DUMC 3094, Durham, NC 27710 USA; 2 Department of Surgery, Duke University, Durham, USA

**Keywords:** Enhanced recovery, Goal-directed fluid therapy, Perioperative acute kidney injury, RIFLE criteria

## Abstract

**Background:**

While enhanced recovery protocols (ERPs) reduce physiologic stress and improve outcomes in general, their effects on postoperative renal function have not been directly studied.

**Methods:**

Patients undergoing major colorectal surgery under ERP (February 2010 to March 2013) were compared with a traditional care control group (October 2004 October 2007) at a single institution. Multivariable regression models examined the association of ERP with postoperative creatinine changes and incidence of postoperative acute kidney dysfunction (based on the Risk, Injury, Failure, Loss, and End-stage renal disease criteria).

**Results:**

Included were 1054 patients: 590 patients underwent surgery with ERP and 464 patients without ERP. Patient demographics were not significantly different. Higher rates of neoplastic and inflammatory bowel disease surgical indications were found in the ERP group (81 vs. 74%, *p* = 0.045). Patients in the ERP group had more comorbidities (ASA ≥ 3) (62 vs. 40%, *p* < 0.001). In unadjusted analysis, postoperative creatinine increase was slightly higher in the ERP group compared with control (median 0.1 vs. 0 mg/dL, *p* < 0.001), but levels of postoperative acute kidney injury were similar in both groups (*p* = 0.998). After adjustment with multivariable regression, postoperative changes in creatinine were similar in ERP vs. control (*p* = 0.25).

**Conclusions:**

ERP in colorectal surgery is not associated with a clinically significant increase in postoperative creatinine or incidence of postoperative kidney injury. Our results support the safety of ERPs in colorectal surgery and may promote expanding implementation of these protocols.

**Trial registration:**

Not applicable, prospective data collection and retrospective chart review only.

## Background

Enhanced recovery protocols (ERPs) are multimodal approaches focusing on improving patient surgical outcomes through preoperative optimization and emphasis on standardized evidence-based interventions in perioperative patient care. A growing body of evidence suggests that ERPs significantly reduce the incidence of perioperative complications, length of hospitalization, and health care costs for patients undergoing colorectal surgery (Miller et al., 2014; Zhuang et al., 2013; Lv, 2012).

Acute kidney injury (AKI) is a relatively common postoperative complication after colorectal surgery (Masoomi et al., 2012). Although the etiology of AKI following surgery is multifactorial, it has been traditionally thought that liberal fluid administration may be beneficial in the perioperative period, when patients are predisposed to reductions in renal blood flow (Fearon et al., 2005; Lyon et al., 2012).

ERPs attempt to avoid fluid overload during both the intraoperative and postoperative periods. Intraoperatively, low-dose maintenance crystalloid infusions are advocated to maintain zero fluid balance. In addition, many centers use goal-directed fluid therapy to optimize stroke volume and deliver fluids only to patients who are volume-responsive, as judged by stroke volume assessment (Miller et al., 2015). In the postoperative period, intravenous fluids are discontinued after resumption of oral fluid intake, most often in the immediate postoperative period (Miller et al., 2014). Permissive oliguria is tolerated and is not necessarily treated with fluid boluses in the absence of other indicators of hypovolemia.

Increased use of neuraxial analgesia is another component of ERPs. This has been shown to improve postoperative pain control and return of gastrointestinal motility (Steinbrook, 1998). At the same time, the sympatholysis produced by epidural analgesia causes arterial vasodilation (Clemente & Carli, 2008). An increased incidence of postoperative hypotension has been observed in patients treated with epidural analgesia under ERPs (Marret et al., 2007; Gupta & Gan, 2016).

Although there is good evidence for the benefits of avoiding fluid overload, concerns have been raised that the more restrictive fluid management approach in ERPs, permissive oliguria, and the increased use of epidural analgesia common to ERPs may increase the risk for postoperative AKI. Unfortunately, there is a scarcity of data examining the impact of ERP on postoperative kidney function after colorectal surgery. While small studies comparing ERPs to traditional care have reported similar rates of acute kidney dysfunction in their enhanced recovery and conventional therapy cohorts, these studies were underpowered to detect changes in individual complications (Huebner et al., 2014; Hübner et al., 2013; Ihedioha et al., 2015). Large studies, meta-analyses, and systematic reviews comparing ERPs to conventional care have not specifically set out to compare renal outcomes from traditional management to ERPs, so these studies are limited by a lack of granularity that precludes inference about the adjusted renal effects of ERPs (Bakker et al., 2015; Gustafsson et al., 2011; Ren et al., 2012; Varadhan et al., 2010; Aarts et al., 2012; ERAS Compliance Group, 2015; Shida et al., 2015; Dhruva Rao et al., 2015; Spanjersberg et al., 2015; Greco et al., 2014; Gillissen et al., 2013; Gravante & Elmussareh, 2012; Rawlinson et al., 2011). Therefore, we sought to examine directly the effects of an ERP on changes in postoperative creatinine levels and the incidence of postoperative AKI following colorectal surgery.

## Methods

### Study cohort

The study included patients undergoing major elective colorectal surgery under ERP (between February 2010 and March 2013) or without ERP (between October 2004 and October 2007) at the Duke University Medical Center. Eligible procedures for inclusion were segmental colectomy, total abdominal colectomy, total abdominal colectomy with end ileostomy, total proctocolectomy with ileoanal pouch, low anterior resection, and abdominoperineal resection. The study included both laparoscopic and open procedures. Procedures were performed by board-certified colorectal surgeons. Patients with preoperative renal dysfunction (defined as creatinine > 1.5) were excluded. The Duke Institutional Review Board approved this study (IRB#: Pro00061780).

### Colorectal ERP

**Fig. 1:**
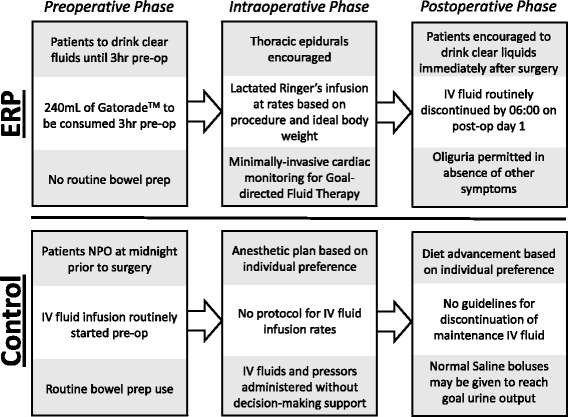
Summary of changes to fluid handling with enhanced recovery protocol (ERP)

Specifics of the Duke ERP and data demonstrating improvements in colorectal surgery outcomes have been published previously (Miller et al., 2014; Adam et al., 2015). Briefly, this protocol was composed of three phases: preoperative, intraoperative, and postoperative. In the preoperative phase, patients received education on the program and details about their role. To minimize preoperative fasting, clear liquids were permitted until 3 h before the time of anesthesia induction. In addition, patients were given a carbohydrate-rich drink 3 h before induction. All patients received standardized preoperative antibiotics and thromboprophylaxis, as well as multimodal strategies for pain management and postoperative nausea and vomiting. Bowel preparation was not routinely employed. In the intraoperative phase, minimally invasive surgical approaches and use of epidural analgesia were encouraged. Ninety-two percent of ERP patients received thoracic epidural analgesia. Maintenance crystalloid therapy was delivered via an infusion pump at 1–3 ml/kg/h. Cardiac output monitors were used to perform goal-directed fluid therapy, with 250 ml boluses of Voluven® (Fresenius Kabi Norge AS, Halden, Norway) given to optimize cardiac output. Esophageal Doppler (EDM™ Deltex Medical, Inc., Irving, TX) was used for non-invasive cardiac output monitoring, and the LiDCORapid™ (LiDCO Ltd., Cambridge, UK) was used if invasive cardiac monitoring was established. During the intraoperative and postoperative periods, oliguria was tolerated if signs or symptoms of hypovolemia were absent. Postoperatively, diet and ambulation were initiated on the day of surgery. Intravenous fluids were most often stopped by 06:00 on postoperative day 1 and only restarted if there were clinical concerns about intolerance of oral intake. The head of the bed was kept at 30°, and epidural anesthesia was continued for up to 72 h following surgery. Figure 1 provides a summary of changes implemented under the ERP that are likely to affect patient fluid balance.

### Data source

**Fig. 2:**
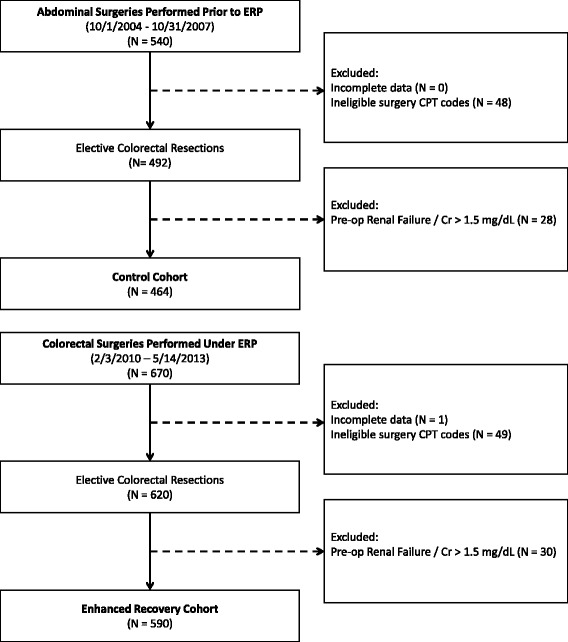
Patient inclusion/exclusion flow diagrams. ERP enhanced recovery protocol, Cr serum creatinine

Data were extracted from two databases—a control cohort, previously identified by review of the Duke Innovian® (Draeger, Inc. Telford PA) perioperative database, and the enhanced recovery cohort, collected in a prospectively maintained database. Patient age, gender, race, American Society of Anesthesiologists (ASA) classification, surgical indication, surgical approach, and extent of surgical resection were extracted from each dataset. Serum creatinine data were collected by the study team via retrospective chart review. Preoperative serum creatinine was defined as the serum creatinine obtained in closest proximity to the date of surgery, usually within 1 week of the procedure. Peak postoperative serum creatinine was defined as the highest creatinine level obtained during the 30 days following surgery. For all patients, the 30-day period allowed for capture of creatinine levels obtained during the inpatient period, follow-up appointment, and readmissions for complications. All patients had a minimum of postoperative day 1 serum creatinine level, pre-discharge serum creatinine level, and follow-up appointment serum creatinine level. Internal auditing was performed to ensure data accuracy. A patient inclusion/exclusion flow diagram is included as Fig. 2.

### Statistical analysis

The primary outcome measure of the study was the incidence of postoperative AKI. AKI was stratified into three classes: No Kidney Injury, Acute Kidney Injury (2× increase in creatinine), and Acute Kidney Failure (3× increase in creatinine). These creatinine change cutoffs correspond to the Acute Dialysis Quality Initiative’s Risk, Injury, Failure, Loss, and End-stage renal disease (RIFLE) classification (Bellomo et al., 2004). Urine output definitions of renal injury were not used because urine output was not strictly tracked in the ERP group, as the protocol calls for early discontinuation of urinary catheters. The risk category was not included, as it does not correspond to actual renal injury.

Relevant patient demographic data, perioperative creatinine, and operative characteristics were compared between the ERP and the pre-implementation (control) groups using Pearson’s chi-square/Fisher’s exact tests for categorical variables. The Wilcoxon rank-sum test was used to compare continuous variables.

Multivariable linear regression modeling was employed to examine the adjusted association between ERP vs. control with changes in postoperative serum creatinine levels while accounting for the effects of patient age, gender, race, ASA score, surgical indication, surgical extent, and surgical approach, and preoperative creatinine level. All statistical analyses were performed using R 3.2.1 (R Foundation for Statistical Computing; Vienna, Austria).

## Results

### Patient and treatment characteristics

A total of 1054 patients were included, 590 (56%) of whom were treated in the ERP group and 464 (44%) patients were in the control group. Patient demographic characteristics were not significantly different between the two groups (Table 1). ASA class tended to be higher in the ERP group (for example, ASA ≥ 3: 62 vs. 40%, *p* < 0.001). The ERP group included fewer colectomies (52 vs. 88%) and more rectal resections (48 vs. 12%) (all *p* < 0.001). Use of laparoscopy was more frequent in the ERP group (60 vs. 49%, *p* < 0.001). Although the total volume of fluid administered intraoperatively was not significantly different between the groups (*p* = 0.233), more colloid was administered in the ERP group (median 500 mL control vs. 1000 mL ERP; *p* < 0.001).

**Table 1:** Patient demographics and treatment characteristics: ERP vs. control

	Control (*N* = 464)	ERP (*N* = 590)	*p* value
Age (years, median [IQR])	60 [51–71]	60 [48–68]	0.175
Sex			0.316
Male	48.9% (227)	52.0% (307)	
Female	51.1% (237)	48.0% (283)	
Race			0.749
White	72.6% (337)	74.7% (441)	
Black	23.5% (109)	21.7% (128)	
Others	3.9% (18)	3.6% (21)	
ASA classification			< 0.001
1	1.7% (8)	3.9% (23)	
2	58.4% (271)	34.4% (203)	
≥ 3	39.9% (185)	61.7% (364)	
Indication			0.045
Benign	25.8% (120)	19.3% (114)	
IBD	11.3% (52)	12.9% (76)	
Neoplastic	62.9% (292)	67.8% (400)	
Extent of surgery			< 0.001
Colectomy	88.1% (409)	52.4% (309)	
Proctectomy	11.9% (55)	47.6% (281)	
Surgical approach			< 0.001
Laparoscopic	48.7% (226)	59.9% (353)	
Open	51.3% (238)	40.1% (237)	
Intra-op total fluid (mL, median [IQR])	3760 [2460–5351]	3468 [2688–4536]	0.233
Intra-op colloid (mL, median [IQR])	500 [0–1000]	1000 [750–1500]	< 0.001
Pre-op hemoglobin (mg/dL, median [IQR])	13.4 [11.8–14.5]	13.3 [11.8–14.5]	0.401

*ERP* enhanced recovery protocol, *ASA* American Society of Anesthesiologists, *IQR* interquartile range, *IBD* inflammatory bowel disease

### Treatment outcomes

**Fig. 3:**
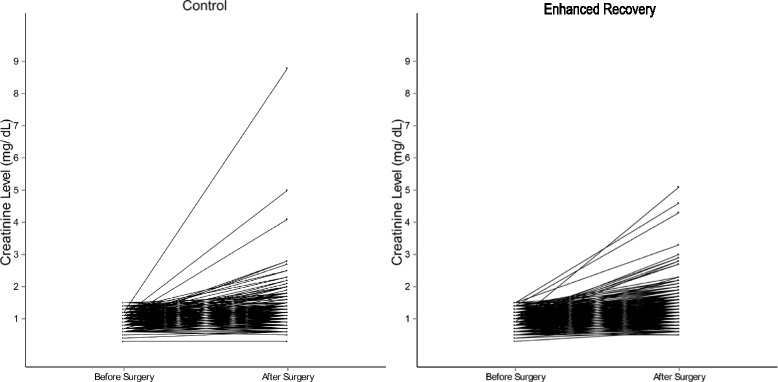
Graphical representation of pre-/postoperative creatinine differences, control and enhanced recovery

Although statistically significant, median serum creatinine levels were not clinically different between groups preoperatively (0.9 vs. 0.9 mg/dL) or postoperatively (1.0 vs. 1.0 mg/dL). However, differences between the preoperative and postoperative serum creatinine levels were slightly higher in the ERP group than in control (median 0.1 vs. 0.0 mg/dL, respectively, *p* < 0.001) (Table 2, Fig. 3).

**Table 2:** Unadjusted renal outcomes in patients treated with ERP vs. traditional care

	Control (*N* = 464)	ERP (*N* = 590)	All patients (*N* = 1054)	*p* value
Preoperative creatinine (mg/dL, median [IQR])	0.9 (0.8–1.1)	0.9 (0.7–1.0)	0.9 (0.8–1.1)	0.002
Max postoperative creatinine (mg/dL, median [IQR])	1.0 (0.8–1.2)	1.0 (0.8–1.2)	1.0 (0.8–1.2)	0.008
Creatinine differences (mg/dL)	0.0 (0.0–0.1)	0.1 (0.0–0.3)	0.1 (0.0–0.2)	< 0.001
Level of postoperative kidney injury				0.998
No kidney injury	95.5% (443)	95.4% (563)	95.4% (1006)	
Acute kidney injury (2× increase)	3.7% (17)	3.7% (22)	3.7% (39)	
Acute kidney failure (3× increase)	0.9% (4)	0.8% (5)	0.9% (9)	

Acute kidney injury and failure thresholds set at 2× and 3× increase in creatinine, based on RIFLE criteria cutoffs. Wilcoxon rank-sum test was used to compare creatinine ranges; Mann–Whitney *U* test was used to compare incidences of kidney injury and failure

*ERP* enhanced recovery protocol, *IQR* interquartile range

Compared with control, patients undergoing surgery in the ERP group had no significant differences in incidence of acute kidney injury (3.7% ERP vs. 3.7%) and acute kidney failure (0.8% ERP vs. 0.9%) (*p* = 0.998).

After adjustment for patient age, gender, race, ASA score, surgical indication, extent of surgery, and surgical approach, ERP vs. control was not associated with significant changes in postoperative serum creatinine levels (*p* = 0.251) (Table 3). Factors associated with significant increases in postoperative serum creatinine were older patient age, male gender, black race, and use of open surgical approach.

**Table 3:** Adjusted associations between ERP and postoperative creatinine differences

Co-variables	Estimated Δ Creatinine	Lower 95% confidence interval	Upper 95% confidence interval	*p* value
ERP vs. control	0.035	− 0.024	0.093	0.251
Increasing age	0.004	0.002	0.005	< 0.001
Female vs. male	− 0.282	− 0.334	− 0.231	< 0.001
Black vs. white	0.089	0.027	0.151	0.005
ASA 2 vs. ≤ 1	− 0.084	− 0.239	0.072	0.293
ASA ≥ 3 vs. ≤ 1	− 0.012	− 0.167	0.143	0.878
IBD vs. benign	0.031	− 0.065	0.127	0.529
Neoplastic vs. benign	− 0.009	− 0.073	0.055	0.784
Proctectomy vs. colectomy	0.031	− 0.032	0.094	0.333
Open vs. laparoscopic approach	0.102	0.047	0.156	< 0.001

## Discussion

This large single-institution study examined the impact of an optimized ERP on perioperative renal function of patients undergoing major colorectal surgery. In unadjusted analysis, patients undergoing surgery within ERP had a small statistically significant increase in postoperative serum creatinine. However, after adjustment for patient and procedure mix, implementation of an ERP was not associated with a statistically significant increase in the levels of postoperative serum creatinine. Further, the incidences of postoperative AKI and acute kidney failure were similar between patients treated with ERP vs. without ERP.

Overall, very few studies have tracked the incidence of renal complications within ERPs. Most individual studies present either a pooled complication rate (overall complications) or a limited subset of individual postoperative complications, which did not include acute kidney injury or failure. Similarly, meta-analyses and systematic reviews of ERPs for colorectal surgery report only classifications of “major” and “minor” complications (Huebner et al., 2014; Hübner et al., 2013; Ihedioha et al., 2015; Bakker et al., 2015; Gustafsson et al., 2011; Ren et al., 2012; Varadhan et al., 2010; Aarts et al., 2012; ERAS Compliance Group, 2015; Shida et al., 2015; Dhruva Rao et al., 2015; Spanjersberg et al., 2015; Greco et al., 2014; Gillissen et al., 2013; Gravante & Elmussareh, 2012; Rawlinson et al., 2011).

In one study, 268 patients undergoing resection with established care were compared with 78 patients treated within an ERP. The rates of acute renal failure were reported as 2.2% in the established care group and 3.8% in the ERP group, which were not significantly different between the two groups (*p* = 0.37) (Huebner et al., 2014). While that smaller study showed equivalent renal outcomes between the ERP and traditional pathway, it was not clear how renal function and/or failure were defined. Another limitation of that study was the small size that precluded adjustment for possible confounders that have known effects on renal function, such as patient age, comorbidities, extent of surgery, and operative technique. A small-cohort review study from the Mayo Clinic compared postoperative hemodynamics in patients who received treatment within an ERP with or without intrathecal analgesia. The study reported a 2.5% incidence of renal failure among its 163 patients (Hübner et al., 2013). That study used the Acute Kidney Injury Network criteria for renal injury and tracked creatinine and urine output, but it was still limited by small cohort size and the lack of a control group.

In our study, after adjustment for possible confounding factors, the rates of acute kidney injury or failure were similar between the ERP and traditional care groups. Our rates of acute kidney failure were lower than the rates reported in the studies discussed above (0.9% vs. 2–4%). This may be due to differences in definitions of acute renal failure, as well as the effect of the small sample sizes in the earlier studies. We utilized the RIFLE criteria to help define postoperative renal function. The RIFLE criteria were initially proposed as a standardized definition of kidney dysfunction to permit for better comparison of studies: these criteria have been validated as having clinical and prognostic significance (Lopes & Jorge, 2013). Basing our creatinine change cutoffs on this validated classification system improves the objectivity of our end-points, as well as the generalizability and clinical significance of our results.

Limitations of our investigation include those that are common to retrospective studies, such as the potential for selection bias. After 2010, all patients undergoing elective colorectal surgery at Duke University Hospital were treated in accordance with the hospital’s enhanced recovery pathway and included in a prospectively maintained database. To improve similarity between the datasets, patients in the pre-ERP cohort who underwent multiple intra-abdominal procedures and emergency colorectal surgeries were excluded. The baseline characteristics of the patients in the control and ERP cohorts were comparable, and we performed multivariable adjustment to account for possible confounders.

An important limitation specific to this study is that the control and ERP groups are separated by time. As a result, there are several institutional variables that were not controlled in our analysis, such as changes in equipment, perioperative staff, and trainees involved in cases. There was only one lead surgeon who oversaw cases in both groups, and lead surgeon was not one of the factors included in our multivariable analysis. As a single institution study, this analysis benefited from greater standardization in fluid handling and perioperative patient care protocols, but our results may have less generalizability.

Hydroxyethyl starch (HES) was used during procedures in both groups. HES exposure was not included in the multivariable adjustment. More colloid was used intraoperatively in the ERP cohort, and all ERP patients received at least 250 mL of HES on incision as part of the goal-directed fluid therapy protocol. The maximum dose of HES in the ERP protocol was 50 mL/kg. As usage of synthetic colloid solutions has been linked to increased incidences of renal failure, it is possible that the protocolized usage of HES affected the observed incidence of renal complications in the ERP cohort (Brunkhorst et al., 2008; Schortgen et al., 2001).

In our analysis, the extent of surgery (e.g., partial colectomy vs. total abdominal colectomy) tended to be greater and the ASA scores tended to be higher in the ERP cohort, so remaining dissimilarities between the pre-ERP and ERP datasets would likely overestimate the ERP’s impact on postoperative kidney injury. Additionally, it is possible that the enhanced recovery cohort captured incidences of renal dysfunction more stringently, as the introduction of this protocol also included resident education to order labs more judiciously, so that patients who appeared to be doing well clinically would be less likely to have had their serum creatinine tracked. Urine output was not as rigorously tracked for patients following ERP, so the urine output criteria for RIFLE classification were not used. The classification of surgical extent and approach was based on CPT codes, which may not accurately reflect the intraoperative techniques and decision-making.

In conclusion, this study evaluated patients undergoing colorectal surgery to assess the potential deleterious effect of enhanced recovery protocol on renal function. By tracking subclinical changes in creatinine, controlling for potential confounders in a diverse population, and employing large patient cohorts to better detect small differences in rates of perioperative AKI, our review rigorously demonstrates that the historical presumptions about the risk of kidney failure with enhanced recovery schemes in colorectal surgery are unfounded in patients without preexisting kidney disease. Given the increasing application of enhanced recovery principles to other surgical specialties, our findings also provide valuable information with regard to the safety of ERPs.

## Conclusions

This study is the first and largest examining the adjusted effect of enhanced recovery principles and intentional fluid management versus traditional care on changes in postoperative creatinine and the incidence of postoperative AKI in colorectal surgery. While clinically insignificant changes in creatinine were observed in unadjusted analysis, the application of enhanced recovery protocols in our colorectal surgery population was not significantly associated with changes in creatinine after adjusting for patient and procedure characteristics. Using outcomes stratified with creatinine cutoffs from the RIFLE criteria, we showed no difference in rates of acute kidney injury or failure between traditional and enhanced recovery perioperative management. As such, intentional fluid management strategies, as part of an enhanced recovery protocol, do not appear to increase the risk of postoperative acute kidney dysfunction.

## Abbreviations

AKI: Acute kidney injury
ASA: American Society of Anesthesiologists
ERP: Enhanced recovery protocol
HES: Hydroxyethyl starch
IBD: Inflammatory bowel disease
RIFLE: Risk, Injury, Failure, Loss, and End-stage renal disease

## References

[CR1] Aarts M-A, Okrainec A, Glicksman A, Pearsall E, Charles Victor J, McLeod RS (2012). Adoption of enhanced recovery after surgery (ERAS) strategies for colorectal surgery at academic teaching hospitals and impact on total length of hospital stay. Surg Endosc.

[CR2] Adam MA, Lee LM, Kim J, Shenoi M, Mallipeddi M, Aziz H, et al. Alvimopan provides additional improvement in outcomes and cost savings in enhanced recovery colorectal surgery*.* Ann Surg. 2015 Oct 22; [Epub ahead of print]. Accessed on 16 Mar 2016. Available at: http://journals.lww.com/annalsofsurgery/Citation/2016/07000/Alvimopan_Provides_Additional_Improvement_in.23.aspx 10.1097/SLA.0000000000001428 26501697

[CR3] Bakker N, Cakir H, Doodeman HJ, Houdijk APJ (2015). Eight years of experience with enhanced recovery after surgery in patients with colon cancer: impact of measures to improve adherence. Surgery.

[CR4] Bellomo R, Ronco C, Kellum JA, Mehta RL, Palevsky P (2004). Acute renal failure—definition, outcome measures, animal models, fluid therapy and information technology needs: the second international consensus conference of the Acute Dialysis Quality Initiative (ADQI) group. Crit Care.

[CR5] Brunkhorst FM, Engel C, Bloos F, Meier-Hellmann A, Ragaller M, Weiler N, Moerer O, Gruendling M, Oppert M, Grond S (2008). Intensive insulin therapy and pentastarch resuscitation in severe sepsis. NEJM.

[CR6] Clemente A, Carli F (2008). The physiological effects of thoracic epidural anesthesia and analgesia on the cardiovascular, respiratory and gastrointestinal systems. Minerva Anestesiol.

[CR7] Dhruva Rao PK, Howells S, Haray PN (2015). Does an enhanced recovery programme add value to laparoscopic colorectal resections?. Int J Color Dis.

[CR8] ERAS Compliance Group (2015). The impact of enhanced recovery protocol compliance on elective colorectal cancer resection: results from an international registry. Ann Surg.

[CR9] Fearon KCH, Ljungqvist O, Von Meyenfeldt M, Revhaug A, Dejong CHC, Lassen K (2005). Enhanced recovery after surgery: a consensus review of clinical care for patients undergoing colonic resection. Clin Nutr.

[CR10] Gillissen F, Hoff C, Maessen JMC, Winkens B, Teeuwen JHFA, von Meyenfeldt MF (2013). Structured synchronous implementation of an enhanced recovery program in elective colonic surgery in 33 hospitals in the Netherlands. World J Surg.

[CR11] Gravante G, Elmussareh M (2012). Enhanced recovery for colorectal surgery: practical hints, results and future challenges. World J Gastrointest Surg.

[CR12] Greco M, Capretti G, Beretta L, Gemma M, Pecorelli N, Braga M (2014). Enhanced recovery program in colorectal surgery: a meta-analysis of randomized controlled trials. World J Surg.

[CR13] Gupta R, Gan TJ (2016). Peri-operative fluid management to enhance recovery. Anaesthesia.

[CR14] Gustafsson UO, Hausel J, Thorell A, Ljungqvist O, Soop M, Nygren J (2011). Adherence to the enhanced recovery after surgery protocol and outcomes after colorectal cancer surgery. Arch Surg.

[CR15] Hübner M, Lovely JK, Huebner M, Slettedahl SW, Jacob AK, Larson DW (2013). Intrathecal analgesia and restrictive perioperative fluid management within enhanced recovery pathway: hemodynamic implications. J Am Coll Surg.

[CR16] Huebner M, Hübner M, Cima RR, Larson DW (2014). Timing of complications and length of stay after rectal cancer surgery. J Am Coll Surg.

[CR17] Ihedioha U, Esmail F, Lloyd G, Miller A, Singh B, Chaudhri S (2015). Enhanced recovery programmes in colorectal surgery are less enhanced later in the week: an observational study. JRSM Open.

[CR18] Lopes JA, Jorge S (2013). The RIFLE and AKIN classifications for acute kidney injury: a critical and comprehensive review. Clin Kidney J.

[CR19] Lv L, Shao Y, Zhou Y (2012). The enhanced recovery after surgery (ERAS) pathway for patients undergoing colorectal surgery: an update of meta-analysis of randomized controlled trials. Int J Color Dis.

[CR20] Lyon A, Payne CJ, MacKay GJ (2012). Enhanced recovery programme in colorectal surgery: does one size fit all?. World J Gastroenterol.

[CR21] Marret E, Remy C, Bonnet F (2007). Postoperative pain forum group. Meta-analysis of epidural analgesia versus parenteral opioid analgesia after colorectal surgery. Br J Surg.

[CR22] Masoomi H, Carmichael JC, Dolich M, Mills S, Ketana N, Pigazzi A, Stamos MJ (2012). Predictive factors of acute renal failure in colon and rectal surgery. Am Surg.

[CR23] Miller TE, Roche AM, Mythen M (2015). Fluid management and goal-directed therapy as an adjunct to Enhanced Recovery After Surgery (ERAS). Can J Anaesth.

[CR24] Miller TE, Thacker JK, White WD, Mantyh C, Migaly J, Jin J (2014). Reduced length of hospital stay in colorectal surgery after implementation of an enhanced recovery protocol. Anesth Analg.

[CR25] Rawlinson A, Kang P, Evans J, Khanna A (2011). A systematic review of enhanced recovery protocols in colorectal surgery. Ann R Coll Surg Engl.

[CR26] Ren L, Zhu D, Wei Y, Pan X, Liang L, Xu J (2012). Enhanced Recovery After Surgery (ERAS) program attenuates stress and accelerates recovery in patients after radical resection for colorectal cancer: a prospective randomized controlled trial. World J Surg.

[CR27] Schortgen F, Lacherade JC, Bruneel F, Cattaneo I, Hemery F, Lemaire F, Brochard L (2001). Effects of hydroxyethylstarch and gelatin on renal function in severe sepsis: a multicentre randomised study. Lancet.

[CR28] Shida D, Tagawa K, Inada K, Nasu K, Seyama Y, Maeshiro T (2015). Enhanced recovery after surgery (ERAS) protocols for colorectal cancer in Japan. BMC Surg.

[CR29] Spanjersberg WR, van Sambeeck JDP, Bremers A, Rosman C, van Laarhoven CJHM (2015). Systematic review and meta-analysis for laparoscopic versus open colon surgery with or without an ERAS programme. Surg Endosc.

[CR30] Steinbrook RA (1998). Epidural anesthesia and gastrointestinal motility. Anesth Analg.

[CR31] Varadhan KK, Neal KR, Dejong CHC, Fearon KCH, Ljungqvist O, Lobo DN (2010). The enhanced recovery after surgery (ERAS) pathway for patients undergoing major elective open colorectal surgery: a meta-analysis of randomized controlled trials. Clin Nutr.

[CR32] Zhuang C-L, Ye X-Z, Zhang X-D, Chen B-C, Yu Z. Enhanced recovery after surgery programs versus traditional care for colorectal surgery. Dis *Colon rectum*. 2013;56(5):667–78. 10.1097/DCR.0b013e3182812842 23575408

